# Genomic and Phenotypic Insights for Toxigenic Clinical *Vibrio cholerae* O141

**DOI:** 10.3201/eid2803.210715

**Published:** 2022-03

**Authors:** Yaovi M.G. Hounmanou, Brandon Sit, Bolutife Fakoya, Matthew K. Waldor, Anders Dalsgaard

**Affiliations:** University of Copenhagen, Frederiksberg, Denmark (Y.M.G. Hounmanou, A. Dalsgaard);; Brigham & Women’s Hospital, Boston, Massachusetts, USA (B. Sit, B. Fakoya, M.K. Waldor);; Harvard Medical School, Boston (B. Sit, B. Fakoya, M.K. Waldor)

**Keywords:** cholera, Vibrio cholerae O141, bacteria, toxigenic, genomics, phenotypes, intestinal colonization, public health, enteric infections, zoonoses, United States, Japan

## Abstract

*Vibrio cholerae* remains a major public health threat worldwide, causing millions of cholera cases each year. Although much is known about the evolution and pathogenicity of the O1/O139 serogroups of *V. cholerae*, information is lacking on the molecular epidemiology of non‒O1/O139 strains isolated from patients who have diarrheal illnesses. We performed whole-genome sequence analysis and in vivo infections to investigate characteristics of *V. cholerae* O141 isolated from sporadic diarrheal cases in 4 countries. The strains formed a distinct phylogenetic clade distinguishable from other serogroups and a unique multilocus sequence type 42, but interstrain variation suggests that O141 isolates are not clonal. These isolates encode virulence factors including cholera toxin and the toxin-coregulated pilus, as well as a type 3 secretion system. They had widely variable capacities for intestinal colonization in the infant mouse model. We propose that O141 isolates comprise a distinct clade of *V. cholerae* non‒O1/O139, and their continued surveillance is warranted.

There are an estimated 3–4 million cases of cholera globally each year, driving marked interest in understanding the genomic diversity and evolution of the causative pathogen (*1*,*2*). Of the >200 known *Vibrio cholerae* serogroups distinguished by unique O-antigen structures, only O1 and O139 have been recognized as being capable of causing sustained epidemics. The O139 serogroup, which caused large epidemics on the Indian subcontinent during 1992–1994, arose from *V. cholerae* O1 by exchange of the O139 gene cluster encoding O-antigen biosynthesis for the O1 cluster (*3*). The 2 biotypes of *V. cholerae* serogroup O1 have been the causes of the previous 6 (classical) and ongoing seventh cholera pandemic (El Tor) (*4*,*5*). Decades of study of *V. cholerae* O1 have showed that cholera pathogenesis is largely driven by the activity of the secreted cholera toxin (Ctx), a potent AB_5_ toxin that targets intestinal epithelial cells and causes secretory diarrhea in infected hosts. *V. cholerae* intestinal colonization depends on the toxin-coregulated pilus (Tcp), which is coordinately expressed with Ctx (*6*).

Compared with information available on *V. cholerae* O1, relatively little knowledge is available on the pathogenesis and genomic diversity of *V. cholerae* isolates from other serogroups, such as O37, O75, and O141 (collectively termed non‒O1/O139). These serogroups have been isolated from patients who had diarrheal illness, as well as from aquatic environmental sources (*7*–*10*). In the United States, for instance, toxigenic *V. cholerae* O141 has occasionally been associated with diarrhea and bloodstream infections (*11*,*12*). Although non‒O1/O139 strains can encode Ctx and Tcp, they may be underreported as a cause of diarrheal illness because routine laboratory testing in cholera-endemic settings only includes testing for O1 and O139 serogroups (*13*). Surveillance of non‒O1/O139 serogroups in the United States over the past 30 years has reported diarrheal illness associated with *V. cholerae* O75 and O141 infection from consumption of seafood or exposure to water in lakes and rivers (*8*,*9*).

Previous studies showed that *V. cholerae* O141 isolates can encode Ctx and Tcp (*10*,*14*). In this study, we investigated the genomics and in vivo colonization ability of *V. cholerae* O141 strains isolated from diarrheal cases from 4 different countries during 1984‒1994. The strains were isolated from sporadic cases of diarrhea without any documented epidemiologic association.

## Materials and Methods

### Strain Collection, DNA Extraction, and Whole-Genome Sequencing

We obtained *V. cholerae* O141 isolates sequenced in the present study from a strain collection initially reported by Dalsgaard et al. (*10*,*15*). The strains were isolated from sporadic cases of diarrhea, which did not appear to be epidemiologically related. Information about whether stool samples were cultured for major enteric pathogens other than *V. cholerae* was not available for the strains studied.

We obtained strains from the Center for Disease Control and Prevention (Atlanta, GA, USA) and the Japanese National Institute of Infectious Diseases (Tokyo, Japan). We stored strains in 10% glycerol at −80°C, and revived them by streaking onto blood agar plates. We extracted genomic DNA from overnight liquid cultures of the isolates by using the Maxwell RSC Cultured Cells DNA kit following the manufacturer’s protocol and the automated Maxwell RSC Machine (both from Promega, https://www.promega.com). We sequenced genomic DNA samples by using the MiSeq System (Illumina, https://www.illumina.com) as described (*16*). The coverage of the sequenced genomes ranged from 50× to 75× (Table). We submitted the sequence reads to the European Nucleotide Archive (accession no. PRJEB42289).

**Table:** Characteristics of whole-genome sequences of *Vibrio cholerae* O141 strains*

Strain	GC, %	No. contigs	Length, bp	N50 of contigs	Place and year of isolation	cgMLST†
AD3_609–84	47.5	136	3,959,387	195,630	USA, 1984	479
AD4_2454–85	47.44	145	4,110,364	104,771	USA, 1985	479
AD5_2466–85	47.42	134	4,096,622	111,701	USA, 1985	479
AD6_2527–87	47.52	140	4,073,408	111,688	USA, 1987	479
AD7_2533–86	47.41	150	4,056,508	157,587	USA, 1986	479
AD8_F2031	47.43	101	3,976,610	187,293	Spain, 1994	246
AD9_234–93	47.5	130	4,046,144	185,481	India, 1993	479
AD10_1178–96	47.41	118	4,082,579	101,628	Taiwan, 1993	479

*cgMLST, core genome multilocus sequence type; N50, shortest contig length covering 50% of the genome. †The conventional 7-gene MLST profile is ST42 for all.

### Read Processing and Genome Assembly

We trimmed raw sequence reads by using with bbduk2 (*17*) (from BBmap version 6.49) and a cutoff score of 20. We evaluated read quality by using FastQC version 0.11.5 (https://guix.gnu.org) before and after trimming. We assembled trimmed reads by using Spades version 3.13.0 (*18*), error correction, a coverage cutoff of 2, and kmer sizes 21, 33, 55, 77, 99 and 127. We discarded contigs <200 bases and assessed the quality of the de novo assembled contigs by using Quast version 4.5 (*19*). We then analyzed the assembled genomes for species identification and *V. cholerae*–specific genome annotation (biotype, serogroup, and *Vibrio* pathogenicity island conservation) by using the CholeraeFinder tool (https://cge.cbs.dtu.dk/services/CholeraeFinder). We identified resistance genes by using ResFinder (20) and plasmid replicons by using PlasmidFinder (*21*).

### Phylogenetic Analysis

We used the generated *V. cholerae* O141 genomes for phylogenetic analysis with publicly available genomes representing the other Ctx-positive *V. cholerae* serogroups. Representative clinical nontoxigenic and non‒O1/O139 genomes from strains isolated in Germany were also included in the analysis (*22*). We analyzed 23 additional *ctxA-*positive *V. cholerae* and 7 *ctx*A-negative non‒O1/0139 reference genomes and compared them with the 8 genomes we had (total = 38). These genomes included the only whole genomes sequences of *V. cholerae* O141 available before this study (strain V51 and 234–93), all publicly available genomes of *V. cholerae* O75 and O37 (all *ctxA+* non‒O1/O139 serogroups), the representative O139 strain MO10, and a variety of historical and contemporary O1 strains with differing *ctxB* alleles, which were selected to capture the genomic variation of pandemic *V. cholerae* O1. These historical and contemporary O1 strains included strains O395 (classical, *ctx*B1), N16961 (El Tor, *ctx*B3), CTMA1422 (El Tor variant, *ctx*B1), L254 (El Tor variant, *ctx*B1) and ZB6 (El Tor variant, *ctx*B7). We provide details and accession numbers of these genomes, including the nontoxigenic non‒O1/O139 strains (Appendix Table 1).

We called single-nucleotide variants by using Snippy version 4.6.0 (https://github.com/tseemann/snippy) under the following parameters: mapping quality of 60, a minimum base quality of 13, a minimum read coverage of 4, and a 75% concordance at a locus. We aligned core genome single-nucleotide variants by using Snippy version 4.1.0 for phylogeny inference. We detected masked putative recombinogenic regions by using Gubbins version 2.4.1 (*23*). We built a maximum-likelihood phylogenetic tree by using RAxML version/8.2.12 and the generalized time-reversible model with 100 bootstraps (*24*). We rooted the final tree on the V51 genome and visualized it with iTOL version 3 (*25*). We provide pairwise single-nucleotide polymorphism (SNP) data for the 38 strains (Appendix Table 2). The alignment length from all analyzed genomes was 3,464,958 and represented 82.3% of the reference *V. cholerae* strain V51 used.

### Comparative Genomics

We annotated all genomes used for phylogenetic analysis by using Prokka version 1.14.5 (*26*), and used resulting general feature format 3 files as inputs to the Roary version 3.7.0 (*27*) pangenome analysis tool. We then used the binary presence/absence data of the accessory genome produced in Roary to calculate associations between all genes in the accessory genome and serogroups by using Scoary version 1.6.11 (*28*). We depicted a heatmap of the genes present or absent in the core genome, along with the accessory genome, in phandango (*29*) to enable the identification and extraction of the unique coding sequence (CDS) blocks observed for the O141 serogroup by applying the query_pan_genome function of Roary. After a BLAST Atlas analysis from the GView server (https://server.gview.ca), we mapped the multi-FASTA files of the O141-specific CDS block to the reference V51 to localize the block in the genome.

To understand how O141-specific CDS could play a role in intestinal colonization, we analyzed the extracted multi-FASTA file by using the VRprofile pipeline (*30*), which detects virulence and colonization determinants within bacterial genomes. We customized this analysis to focus on the gene clusters encoding Tcp, T3SS2 a *Vibrio* type III secretion system that is found in clinical *V. parahaemolyticus* isolates and in some *V. cholerae* non‒O1/O139, and other accessory colonization factors known to promote *V. cholerae* intestinal colonization (*31*–*34*). In addition, we individually investigated genes/open reading frames located in these clusters by using local blastn and blastp (https://blast.ncbi.nlm.nih.gov/Blast.cgi) searches against our query genomes with intentionally low 60% query cover and 30% identity thresholds to avoid false-negative gene, absence outcomes that might be caused by recombination.

### Infant Mouse Intestinal Colonization Assay

We orally inoculated 5-day-old, infant CD-1 mice (Charles River Laboratories https://www.criver.com) with *V. cholerae* as described (*35*). We used frozen stocks of each strain to inoculate lysogeny broth that did not contain antimicrobial dugs and incubated the broth overnight with shaking at 250 rpm at 37°C. We diluted cultures 1:1,000 in lysogeny broth and mixed the cultures with 4 μL/mL of green food coloring to track the inoculum. We removed pups from their dams 1 hour preinoculation and orally inoculated them with 50 μL of diluted culture (≈2–4 ×10^5^ CFU/pup). We combined and randomly assigned pups from multiple litters to inoculation groups to reduce the effect of litter effects on *V. cholerae* colonization. We housed inoculated pups in a warmed box with nest material for 20 hours in the dark apart from their dams, at which point they were euthanized with isoflurane inhalation followed by decapitation. We dissected and mechanically homogenized small intestines by using a Tissue Tearor (BioSpec, https://biospec.com), followed by serial dilution and bead plating onto thiosulfate-citrate-bile salt (TCBS) agar plates that did not contain antimicrobial drugs. We incubated plates at 37°C overnight for counting. No non-*Vibrio* (non-yellow) colonies were detected on the TCBS agar plates. Animal work in this study was approved by the Brigham and Women’s Hospital Institutional Animal Care and Use Committee under Protocol #2016N000416.

## Results

### Genomic Characterization and Phylogenetic Analysis

To investigate the genomic diversity of clinical isolates of *V. cholerae* O141, we sequenced and annotated the genomes of 8 serotype-confirmed O141 strains collected from stool samples of gastroenteritis patients in the United States, Spain, Taiwan, and India over a 10-year period during 1984–1994 (*10*) (Table). These strains had been characterized by using ribotyping, PCRs for *ctx*A and *tcp*A, and antimicrobial drug susceptibility testing, but little was known about their genomic characteristics (*10*,*15*). All 8 isolates had gene sequences in the O-antigen lipopolysaccharide region and gene rearrangements between *gmh*D and *wbf*Y, consistent with known O141-specific lipopolysaccharide changes (Appendix Table 3) (*9*,*36*). Sequence typing also placed all 8 isolates in the same multilocus sequence type (MLST), MLST42, as the known O141 isolate V51 (Table). On the basis of concordance in the 7-gene MLST profile, these observations suggest that ST42 might be specific to the serogroup O14, and could serve as a serogroup-specific marker for genomic studies because no other *V. cholerae* serogroups have been associated with this MSLT (*2*,*16*,*37*).

**Figure 1:**
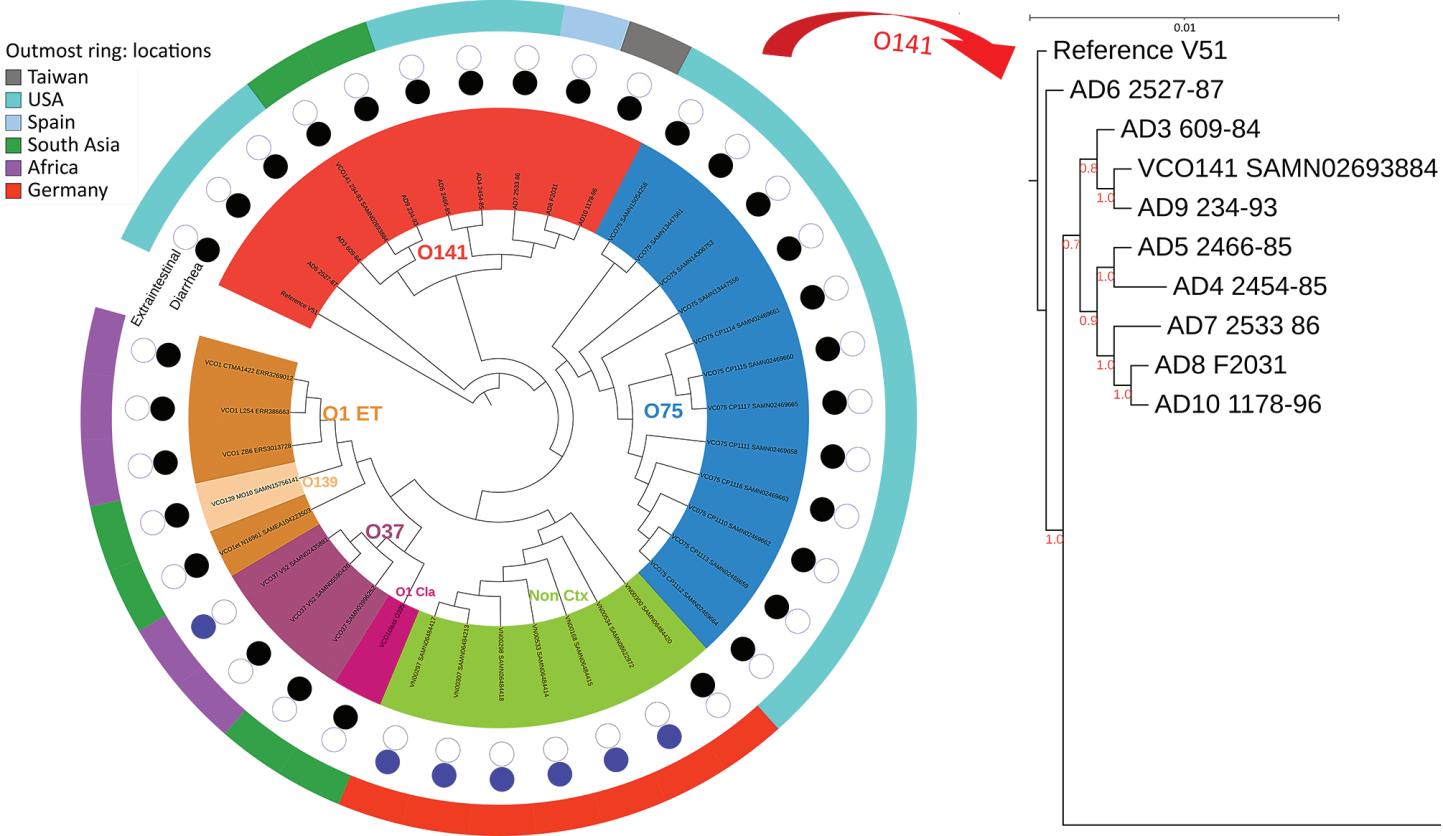
Maximum-likelihood phylogenetic tree for *Vibrio cholerae* O141 in a global context for 38 isolates from Ctx-positive *V. cholerae* and Ctx-negative serogroups. Numbers along branches are bootstrap values. Scale bar indicates nucleotide substitutions per site. Ctx, cholera toxin.

The core genome MLST, which is based on the entire core genome rather than the 7 housekeeping genes used for conventional MLST, was cgST-479 for all except the strains AD8 (cgST-246) and V51 (cgST-248). This variation was further reflected in the whole-genome phylogenetic analysis, in which O141 strains, although distinct from other serogroups, were not internally clonal, differing in up to 261 SNPs (Figure 1).

Despite their diverse sites and years of isolation, all 8 O141 strains encoded a CTX prophage similar to the classical CTX prophage with the *ctxB1* allele and the classical rstR as indicated (*38*). The presence of the classical CTX prophage in all 8 strains suggests that the presence of this sequence along with the alleles that constitute MLST42 might be characteristic of serogroup O141. In addition to *ctxAB*, the genes encoding the signature virulence factor of *V. cholerae*, these strains also encoded cholix toxin, an accessory toxin that is found in *V. cholerae* non‒O1/O139 (*39*). Although these strains harbored a classical CTX prophage, they all also contained an El Tor type *tcpA*, which encodes the major subunit of the Tcp pilus, the CTXϕ receptor, and a critical determinant of intestinal colonization *V. cholerae* O1 (*40*). Although most strains also contained genes in the *tcp* operon needed for Tcp biogenesis (encoded in the *Vibrio* pathogenicity island VPI-I), they generally lacked an intact *tcpJ*, which encodes a prepilin peptidase required for processing of TcpA (*41*). All sequenced strains also appeared to encode a type III secretion system (T3SS) known as T3SS2, that is a critical colonization and virulence determinant of *V. parahaemolyticus* and is also found in V51 (*34*,*42*). The co-occurrence of the TCP and T3SS2 pathogenicity islands in *V. cholerae* O141 strains suggests that *V. cholerae* O141 might rely on diverse mechanisms for pathogenicity, potentially deploying these distinct virulence mechanisms in different hosts.

The O141 strains did not contain detectable antimicrobial resistance genes, supporting prior phenotypic antimicrobial drug susceptibility findings in which all strains were susceptible to a panel of 12 antimicrobial drugs, except for colistin (to which all non‒O1 *V. cholerae* naturally show resistance) (*10*). In addition, none of the analyzed *V. cholerae* O141 genomes contained plasmid replicons, consistent with the absence of plasmids, as shown by previous plasmid extraction analysis of these isolates (*10*).

Despite the observed homogeneity in MLST profile and conservation of major virulence genes in *V. cholerae* O141 strains, there were substantial variations in the O141 genomes (up to 261 SNPs), regardless of country of origin (Figure 1; Appendix Table 2), most of which occurred in noncoding regions. This finding suggests that the strains are epidemiologically unrelated, consistent with the idea that infections caused by *V. cholerae* O141 are sporadic. All the O141 serogroup strains, including V51, formed a separate clade distinguishable from the other serogroups, all strains from serogroup O75 also grouped into a distinct clade (Figure 1). The observed genetic variations between the serogroups indicates that *V. cholerae* O141 and O75 are not phylogenetically related, contrary to a previous proposal (*8*). The phylogeny also suggests that serogroup O37 is closely related to the classical O1 strain O395. As expected, serogroup O139 represented by the reference strain MO10 was localized to the O1 El Tor subclade, consistent with the idea that this serogroup arose from an O1 El Tor seventh pandemic strain (*3*,*21*,*37*). Moreover, the nontoxigenic non‒O1/O139 clinical strains formed a separate clade on the phylogenetic tree that is unrelated to the other known toxigenic, as well as nontoxigenic serogroups.

### Intestinal Colonization of Infant Mice by *V. cholerae* Serogroup O141

**Figure 2:**
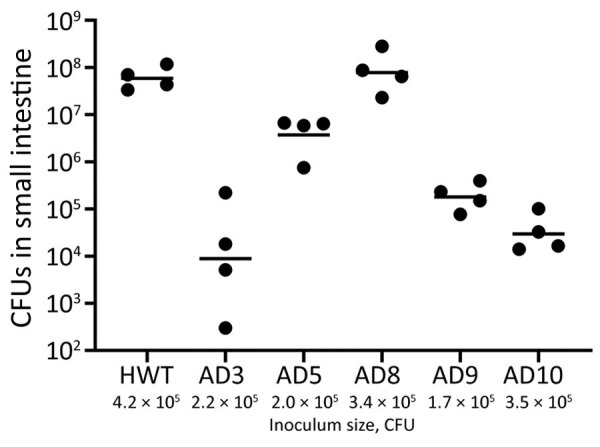
Intestinal colonization of 5-day-old infant mice by *Vibrio cholerae* O141. Pups were orally inoculated with the indicated amount of the indicated strain, and CFUs in the small intestine were enumerated at 20 hours postinoculation. Dots indicate individual animals, and horizontal bars indicate geometric means of each group. HWT, *V. cholerae* O1 isolate from the recent cholera epidemic in Haiti used as a positive control; AD, *V. cholerae* O141 strains analyzed in this study.

The presence of canonical pandemic *V. cholerae* colonization factors such as Tcp in their genomes led us to hypothesize that O141 strains, like their pandemic O1 counterparts, might colonize the small intestine. To test this idea, we used the well-characterized infant mouse model of *V. cholerae* small intestinal colonization. Infant mice orally inoculated with 2–4 × 10^5^ CFU of selected O141 strains that grew well on TCBS agar plates (AD3, AD5, AD8, AD9, and AD10) showed marked variation in their colonization capacity (Figure 2). In comparison to a *V. cholerae* O1 isolate from the recent cholera epidemic in Haiti, which robustly colonizes the small intestine (*43*), strains AD8 and AD5 had similar numbers of CFU recovered in intestinal homogenates as the strain from Haiti (Figure 2). In contrast, the other 3 strains had from ≈1,000-fold (AD9) to ≈10,000-fold (AD3 and AD10) lower numbers of recoverable bacteria, indicating that although they can all colonize the small intestine, there are considerable strain-specific differences in the capacities of these O141 isolates to colonize the mammalian small intestine.

**Figure 3:**
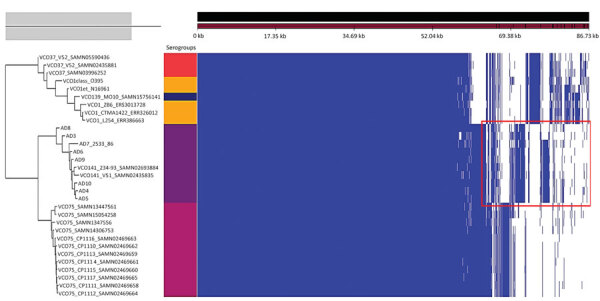
Gene presence/absence map of the pangenome of 31 isolates from cholera toxin‒positive *Vibrio cholerae* serogroups. The red rectangle in the accessory genome indicates the conserved unique coding sequences that are specific to the serogroup O141. Each serogroup is associated with a color block: O37 (red), O1 (orange), O139 (dark blue), O141 (purple), O75 (Marron). Scale bar indicates nucleotide substitutions per site.

**Figure 4:**
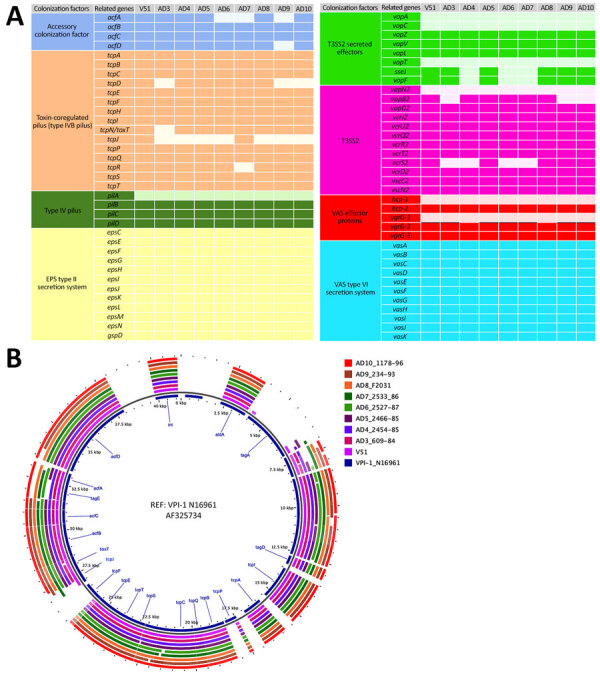
Targeted analysis of the accessory genome of in vivo‒tested *Vibrio cholerae* O141 isolates. A) Genes encoding colonization factors. For each block of colonization factor, the absent genes are represented by the light color. B) Thin gray line after the reference is a standard circle line from the GView server (https://server.gview.ca) delimiting the reference from analyzed samples. EPS, exopolysaccharide; VAS, virulence-associated secretion.

Differential genomic conservation of virulence or colonization determinants could underlie the variable colonization phenotypes. To evaluate strain-level conservation of accessory genetic features, we next performed pangenome analysis of genomes from only the toxigenic strains used in the phylogenetic analysis. This analysis identified an accessory genome made of shell and cloud genes of 2,598 coding sequences (CDS) in a total pangenome size of 5,627 CDS (Figure 3; Appendix Table 4). A targeted analysis of the accessory genome showed strain-specific gene absences in the in vivo‒tested O141 strains (Figure 4). For example, AD3, which had the lowest intestinal colonization among the strains tested, lacked *toxT*, the master transcription activator of *V*. *cholerae* virulence genes (*44*) (Figure 4). The accessory genomes of AD3, AD9, and AD10, which did not colonize as well as the robustly colonizing strains AD5 and AD8, all lacked T3SS2 genes *vcrS2* and *vopB2* (Figure 4, panel A). AD3 also lacked the known T3SS effectors *vop*F and *sse*J (Figure 4, panel A). All analyzed strains, including V51, contained protein sequences corresponding to VopV and VopZ, 2 T3SS2-associated genes known to be critical for intestinal colonization by *V. parahaemolyticus* (*34*,*42*).

## Discussion

Our findings show that *V. cholerae* O141 clinical isolates form a genetically distinct clade that is distinguishable from pandemic and nonpandemic *V. cholerae* serogroups. The observation that all tested isolates encoded known virulence factors and were capable of colonizing the infant mouse intestine, albeit in a highly variable manner, supports the idea that *V. cholerae* O141 could be an underestimated source of cholera-like diarrhea. Currently, O141 cases would be grouped under the umbrella of non‒O1/O139 cases because of a lack of widely available serogroup-specific antiserum for O141. Nevertheless, from this study, the ST42 that appears to be specific/unique to the serogroup O141 might be used for diagnostic purposes as an alternative to O141 antiserum, which is not widely available.

Our findings show that some O141 strains are capable of robust colonization. These strains encode at least 2 potential mechanisms, Tcp and T3SS2, that could enable intestinal colonization. Variable colonization among O141 strains could be explained by differential conservation of T3SS components/effectors or other colonization factors. Deciphering the colonization requirements of different O141 isolates will be a useful endeavor.

The factors that have limited *V. cholerae* O141 from causing sustained cholera epidemics remain to be elucidated. It is possible that *V. cholerae* O141 is not as well adapted as *V. cholerae* O1 to the aquatic environment, which is thought to be a key feature of the lifecycle of *V. cholerae*. Although we did not assess the aquatic fitness of the O141 serogroup, *V. cholerae* O141 has been detected in environmental reservoirs, such as oysters, clams, and freshwater in lakes and rivers in the United States, suggesting an environmental defect is unlikely to fully explain the low frequency of these strains in the clinic (*8*,*9*). These discrepancies call for further genomic and experimental studies on environmental, as well as additional clinical *V. cholerae* O141 isolates. Additional techniques, such as multilocus sequence typing, could overcome challenges related to the identification of *V. cholerae* non‒O1/O139 serogroups.

Overall, *V. cholerae* O141 strains constitute a distinct phylogenetic clade that includes shared and unique genomic elements. In addition, we found that *V. cholerae* O141 clinical isolates showed marked variation in intestinal colonization capacity in the infant mouse model. These findings shed light on a little-known *V. cholerae* serogroup associated with diarrheal illness.

Appendix Additional information on genomic and phenotypic insights for toxigenic clinical *Vibrio cholerae* O141.
